# Staged microsurgical treatment of a complex scalp arteriovenous fistula combined with type C sAVF and cirsoid aneurysm: a case report and literature review

**DOI:** 10.3389/fonc.2024.1452695

**Published:** 2024-12-20

**Authors:** Juncheng Wang, Tao Kuai, Aichao Du, Lihua Yu, Yuzhen Duan, Guoqiang Yuan, Dongming Ma, Yawen Pan

**Affiliations:** ^1^ The Second Clinical Medical School, Lanzhou University, Lanzhou, China; ^2^ Department of Neurosurgery, People’s Hospital of Ningxia Hui Autonomous Region, Yinchuan, China; ^3^ Department of Radiology, People’s Hospital of Ningxia Hui Autonomous Region, Yinchuan, China; ^4^ Department of Function, People’s Hospital of Ningxia Hui Autonomous Region, Yinchuan, China; ^5^ Department of Neurosurgery, The Second Hospital of Lanzhou University, Lanzhou, China; ^6^ Gansu Provincial Key Laboratory of Neurology, Lanzhou, China

**Keywords:** scalp arteriovenous fistula, cirsoid aneurysm, vascular malformation, microsurgery, interventional embolization

## Abstract

Scalp arteriovenous fistula (sAVF) is a rare disease caused by a congenital defect or exogenous injury, but no standard treatment exists. In this article, we report a rare case of sAVF combined with type C sAVF and cirsoid aneurysm (CA), which was successfully treated by staging microsurgery. Individualized surgical incisions were designed based on the size and range of the sAVF, and then staging microsurgery was performed. The first surgery was performed by selectively ligating the supply arteries and fistula of the sAVF. The second surgery was performed by total excision of the vascular malformation a month later. The volume of the vascular malformation in the subcutaneous area decreased after the first surgery, and the vascular malformation in the subcutaneous area was completely removed after the second surgery. In the end, we conclude that the nidus or fistula can be removed entirely through personalized surgical incisions for complicated sAVF combined with type C sAVF and CA.

## 1 Introduction

Scalp arteriovenous fistula (sAVF) is a rare type of scalp vascular malformation caused by congenital, traumatic, iatrogenic, and other factors. Its symptoms and prognosis are extremely variable. Mostly, sAVFs produce a pulsating mass, headache, local pain, bruits, tinnitus, and thrill and are less commonly associated with hemorrhage and necrosis. Digital subtraction angiography (DSA) is the gold standard for its diagnosis and can clearly display the fistula and the location and number of supply arteries and drainage veins (1–4). Surgery is the main treatment for this rare vascular disease and includes microsurgical resection and endovascular embolization (5, 6). Yokouchi et al. classified sAVF into three types: type A, which has a single fistula supplied by a single proximal artery; type B, which has a single fistula supplied by multiple arteries; and type C, which has multiple fistulas accompanied by plexiform supply arteries and main dilated drainage veins. For type A, the primary treatment is interventional embolization through the arterial system; for type B, interventional embolization is performed through the venous system; and for type C, the abovementioned procedures and microsurgery are combined (**Table 1**) (7). Cirsoid aneurysm (CA) is an uncommon type of arteriovenous fistula with more than 200 publicly reported cases worldwide. It presents a serpentine appearance accompanied by subcutaneous pulsation, gradually increasing in volume spontaneously or after trauma, and can eventually be healed after interventional embolization and microsurgical resection (8). This paper describes a case of complex sAVF, including type C sAVF and CA, that was successfully treated with staging microsurgery. In addition, we reviewed the literature on sAVF and discussed the etiology, clinical features, and treatment strategies of complex sAVF.

**Table 1:** Three types of sAVF and their primary treatment.

Type	Main feature	Primary treatment
Type A	Has a single fistula supplied by a single proximal artery	Interventional embolization through the arterial system
Type B	Has a single fistula supplied by multiple arteries	Interventional embolization is performed through the venous system
Type C	Has multiple fistulas accompanied by plexiform supply arteries and main dilated drainage veins	Abovementioned procedures and microsurgery

## 2 Clinical data

The patient is a 22-year-old man. His chief complaint was an egg-sized lump on the right temporal skin.

### 2.1 History of present illness

A soybean-sized lump appeared on the right temporal skin without obvious discomfort after the patient experienced trauma 20 years prior, and it was not treated. In the prior 5 years, the lump gradually increased spontaneously and spread to the skin above the parietal, especially in the prior 3 years. The epidermis of the lump became red, and the temperature was higher than that of the surrounding skin, accompanied by a new lump on the right occipital scalp, the volume of which gradually increased; then, the patient sought medical attention in December 2020.

The patient had no past medical, personal, and family history.

### 2.2 Physical examination

**Figure 1:**
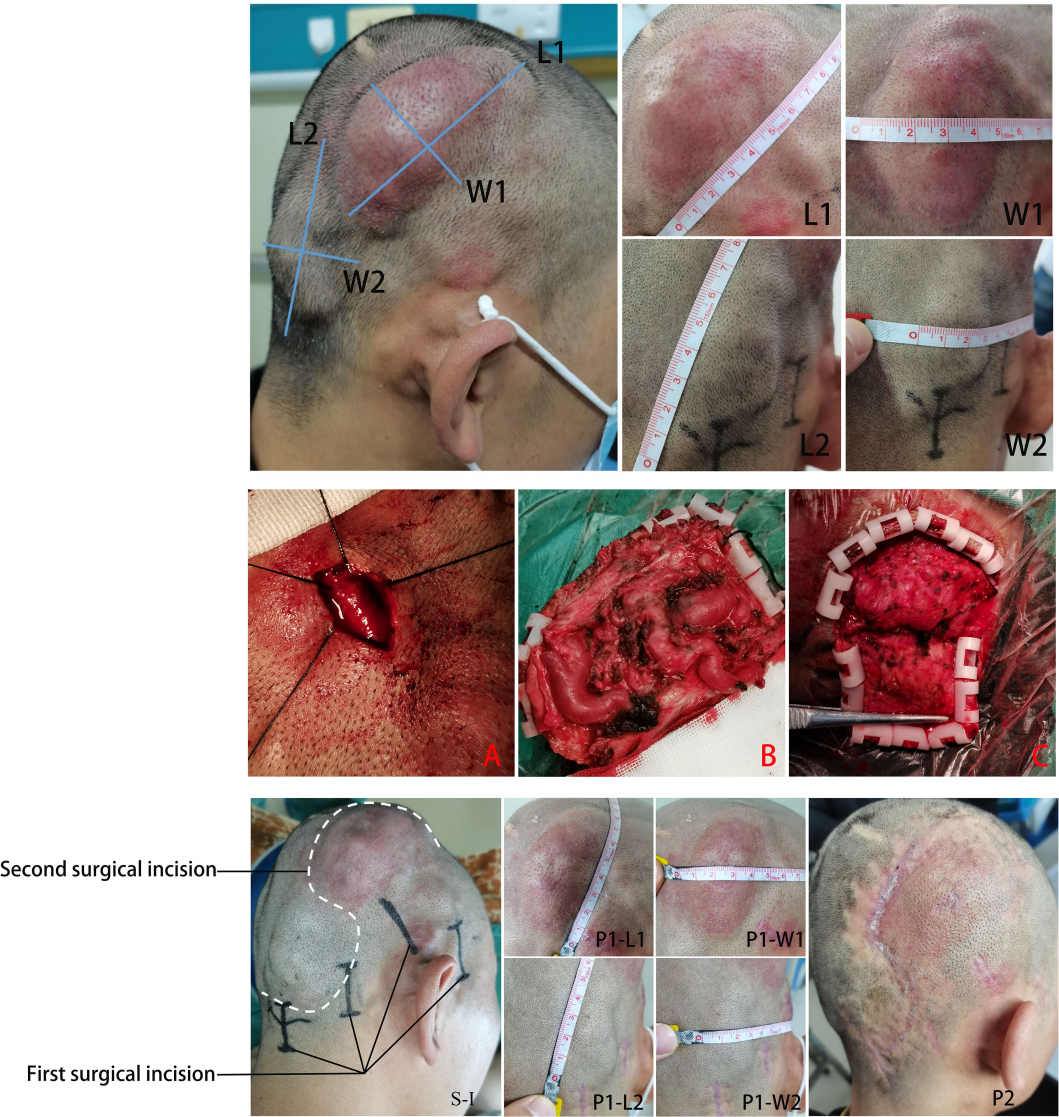
Measurement of the size of the scalp mass (L1/W1, L2/W2). Intraoperative procedures: **(A)** small incisions were used in the first surgery to separate the blood supply artery and drainage vein, and silk suture was used to ligate them; **(B)** large and malformed blood vessels were observed in the right temporal top flap during the second surgery, which was removed by microsurgery; **(C)** large and malformed blood vessels were also observed in the right occipital flap during the second surgery, which was completely removed by microsurgery. Surgical incision design and postoperative healing.

A lump approximately 9.0 cm × 5.0 cm in size in the right temporal lobe with a reddened epidermis and obvious hair loss in some areas and also a lump approximately 7.5 cm × 3.5 cm in size in the right occipital area, which were in high tension and could be reduced by compression, were found in the patient. Obvious blood flow pulsation could be heard during auscultation and palpation (**Figure 1**).

### 2.3 Laboratory and imaging examinations and diagnosis

**Figure 2:**
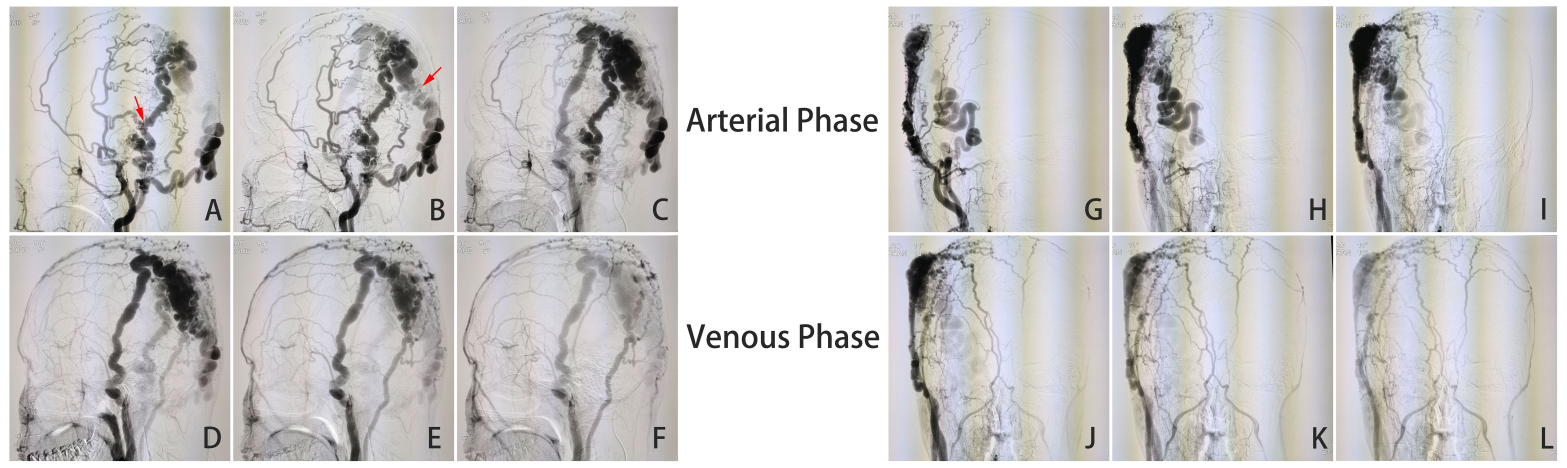
**(A–L)** DSA examination results (arrows show the two main fistulas).

**Figure 3:**
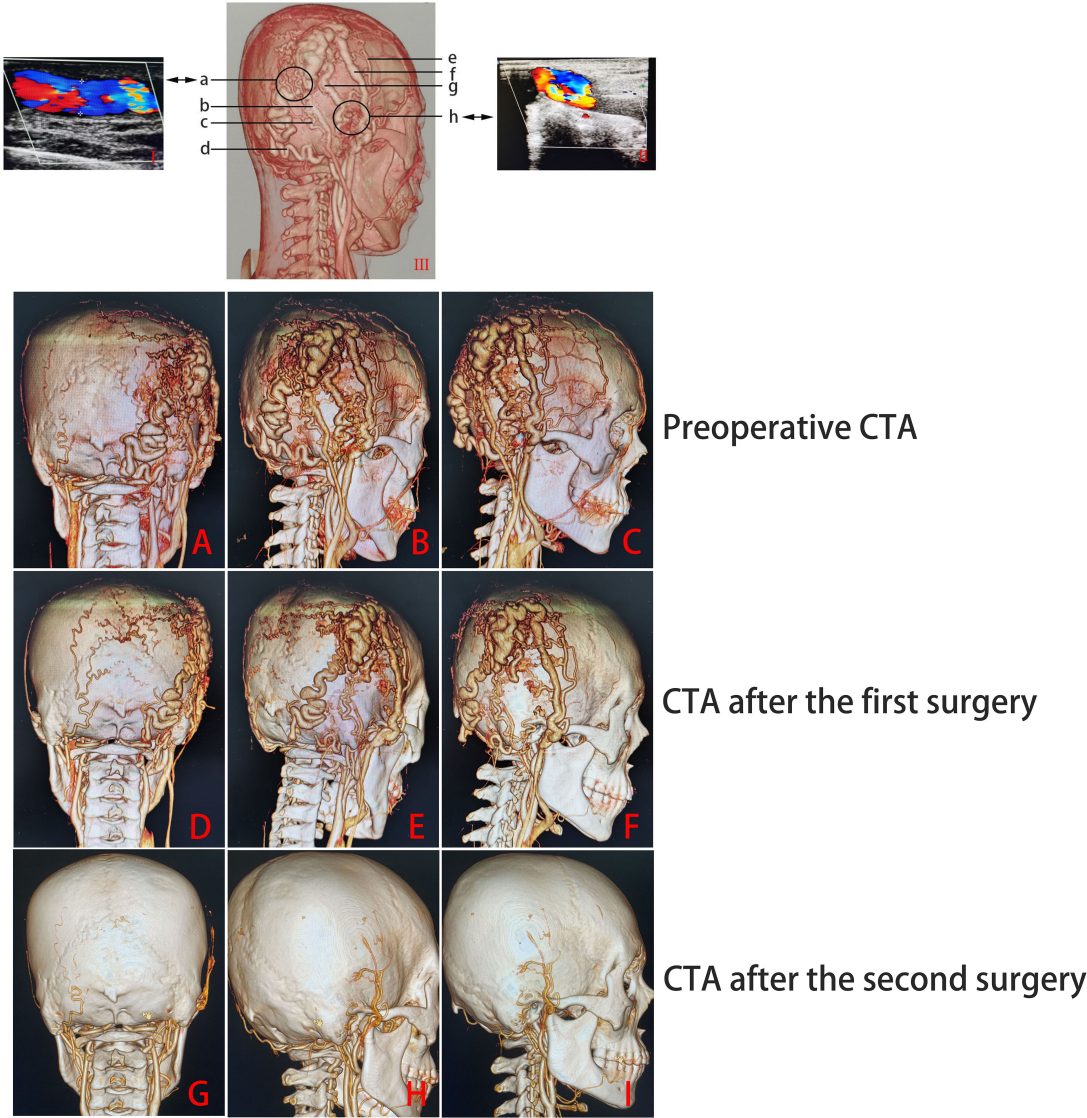
CTA and color Doppler ultrasound examination results (I/II, the results of color Doppler ultrasound; III, the results of the CTA examination; a: the fistula is related to the occipital artery; b, posterior auricular vein; c, posterior auricular artery; d, occipital artery; e, superficial temporal artery; f, superficial temporal vein; g, supplying artery; h, the fistula is related to the superficial temporal artery). Preoperative **(A–C)** and postoperative [first surgery **(D–F)**; second surgery **(G–I)**] CTA examination results.

**Figure 4:**
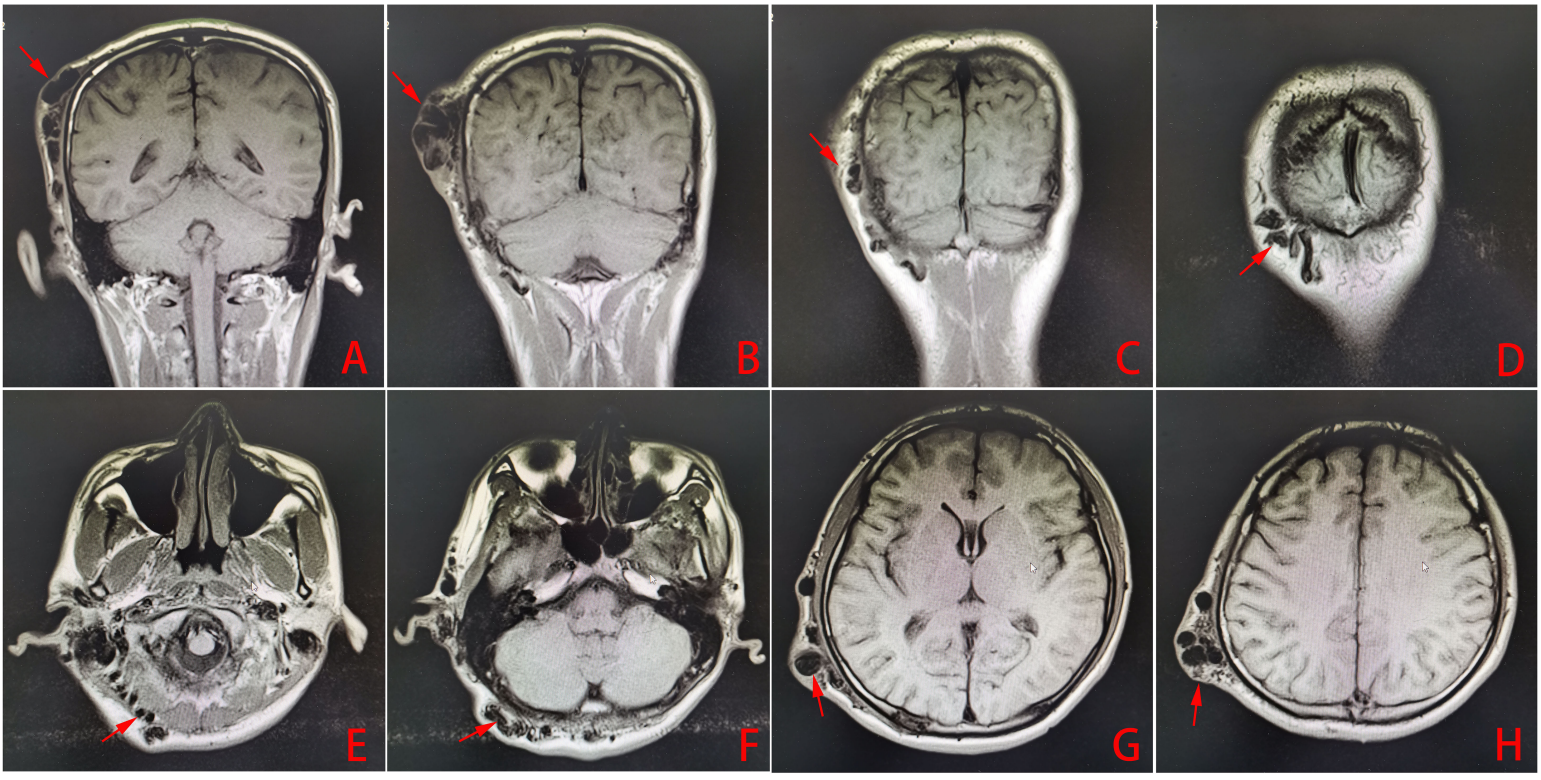
**(A–H)** Head MRI examination results (the arrows show the abnormal vascular mass of the scalp arteriovenous fistula).

No significant difference was found in the patient on lab examination. However, the DSA and CTA examinations revealed a large malformation of blood vessels under the scalp of the right temporoparietal region and a serpentine arrangement of malformation of blood vessels in the right occipital region. The main blood supply vessels for the malformation were the arterialized fistula between the right superficial temporal artery and posterior auricular artery, occipital artery, superficial temporal artery, and posterior auricular artery. The superficial temporal and posterior auricular veins were the main drainage veins, and there was no communication between the scalp malformation of blood vessels and intracranial vessels. Color Doppler ultrasound examination revealed two main fistulas related to the superficial temporal artery and occipital artery (**Figures 2**, **3**). MRI examination showed that there were no other vascular malformations or space-occupying lesions in the brain, showing that the mass was limited to the scalp tissue (**Figure 4**). Based on the examinations conducted, the diagnosis was sAVF.

## 3 Treatment methods and results

### 3.1 Treatment methods

Due to the high cost of endovascular embolization and the poor financial situation of the patient, as well as to the wide range of lesions, complex blood supply arteries and drainage veins, difficulty in selecting microcatheters in place, and fast local blood flow velocity, microsurgical resection was chosen to completely treat and reduce the risk of complications and recurrence in this case. Because the local skin of the patient’s right temporoparietal mass was thin, which was prone to rupture, and considering the possibility of ischemia and necrosis of the local scalp after complete surgical resection, the staged treatment method was adopted: the first surgery was performed by selectively ligating the blood supply artery, drainage vein, and arteriovenous fistula around the mass under local anesthesia (as shown in **Figures 3B–D, G, H**; the surgical incision design is shown in **Figure 1**: S-I), and the second surgery was performed 1 month later to completely remove the subscalp vascular malformation.

### 3.2 Treatment results

In the first surgery, a small incision was used to dissect the supply artery, drainage vein, and arteriovenous fistula and then block the blood flow with suture ligation (**Figure 1**). The size of the mass in the right temporal top scalp was reduced to approximately 8.0 cm × 4.5 cm, and the mass in the right occipital scalp was reduced to approximately 5.0 cm × 3.0 cm 1 month later. The skin color of the right temporal mass was close to normal (**Figure 1**: P1-L1, P1-W1, P1-L2, P1-W2). Cranial CTA showed that the volume of the two subcutaneous vascular malformations of the right temporal and occipital scalp decreased significantly compared to before (**Figures 3A–F**).

The second surgery was performed through an “S”-shaped incision. Large and deformed blood vessels under the skin were visible after flipping the skin flap on the right temporal (**Figure 1B**) and occipital (**Figure 1C**) areas, and the tumor was completely removed under microsurgery. One month after surgery, a follow-up examination showed that the skin incision had healed well, the skin color was normal, and there were no locally palpable masses or abnormal vascular pulsation (**Figure 1**: P2). The CTA of the skull showed that the subcutaneous vascular malformations in the right temporal and occipital region were completely removed (**Figures 3G–I**).

### 3.3 Curative effect and follow-up

The clinical symptoms combined with the imaging examination results were used to evaluate the curative effect, and outpatient visits or telephone calls were conducted to understand the occurrence of postoperative symptoms and complications. Due to the patient’s financial difficulties, the patient did not come to the hospital for follow-up imaging examinations. The patient was followed up twice by phone (with an average of 12 months between follow-ups) more than 2 years after surgery, and in all the follow-ups, the patient reported that the scalp was generally in good condition and that there was no recurrence of the lesion, which was considered a cure.

## 4 Discussion

Scalp AVFs are lesions reported only rarely in the literature, consisting of case reports or small case series. These lesions present a challenge because of their heterogeneous angioarchitecture and non-uniform structure. Thus far, there are no clear guidelines for the treatment of scalp AVFs. In the literature, treatment varies by the type of lesion and is also guided by the physician’s preference (9). The prognosis for an sAVF is extremely variable, and the decision to treat it is based on the patient’s symptoms and risk for exsanguinating hemorrhage. Neurosurgical approaches include ligation of the feeding arteries, surgical resection, electrothrombosis, direct intralesional injection of sclerosing agents, and endovascular embolization. Endovascular intervention is increasingly utilized as a primary treatment or as a preoperative adjunct to surgery (10, 11). Endovascular embolization can reduce the volume of the lesion, thereby improving the related complications. However, it cannot achieve the cosmetic effect of completely resecting the lesion and has a high rate of recurrence. Surgical resection can lead to the complete disappearance of the lesions with a low recurrence rate, and good cosmetic results can be obtained through cosmetic repair. Another important advantage of the operation is that sAVF can be cured through single or staged surgery, such as intravascular embolization, percutaneous embolization, proximal arterial occlusion, and percutaneous blood supply vessel ligation, before or during surgery (12).

Due to the complexity of multiple supply arteries and drainage veins, Ni et al. believe that a suitable individualized treatment plan for C-type sAVF should be developed after evaluation based on the patient’s lesion and the surgeon’s professional technical level (9). Janapareddy et al. reported a typical case of CA, where the scalp showed a serpentine appearance with palpable subcutaneous pulsation, which was ultimately cured after interventional embolization combined with microsurgical resection (8). Kumar et al. reported a case of a small CA that was cured by intravascular embolization (13). Albuquerque et al. also reported a case of a large pediatric CA, which was first reduced in volume by the interventional therapy team through multiple methods (percutaneous embolization with coils and endovascular embolization with cyanoacrylate), and then the plastic surgery team removed the entire lesion. Ultimately, satisfactory results were achieved (14).

The patient in this article had a complex sAVF. The nature of the lesion under the scalp between the right parietal and temporal lobe was similar to that of C-type sAVF, and the nature of the lesion under the scalp on the right occipital lobe was similar to that of CA, between which there was fistula communication (**Figure 3A**). The right superficial temporal artery, posterior auricular artery, occipital artery, and arterialized fistula between the superficial temporal artery and the posterior auricular artery (**Figure 3G**) were the main blood supply vessels for the vascular malformations. The right posterior auricular vein and superficial temporal vein were the main drainage vessels, and there were two main fistulas, namely, the junction between the right occipital artery and the posterior auricular artery (**Figure 3A**) and the junction between the branch of the right superficial temporal artery and the vein behind it (**Figure 3H**). The midline side of the right temporoparietal malformation was accompanied by a large number of plexiform dilated supply arteries and drainage veins. The scalp condition on the right temporal–parietal region and the patient’s family economic conditions were poor. Due to high blood velocity in vascular malformations, if endovascular embolization is performed first, it may cause excessive embolism around the vascular malformations, which could lead to complications such as local scalp ischemic necrosis, making it difficult for microsurgical resection in the later stage and causing a significant economic burden on the patient’s family. At the same time, due to the patient’s medical history of more than 20 years, there may be some small and irreversible mutated and malformed blood vessels in the subcutaneous region, especially those not obvious in imaging examination. Even if the volume of the lesion was further reduced after the main fistula (**Figures 3A, H, G**) was completely ligated in the first surgery, the possibility of recurrence of the sAVF would increase in the later period without the residual small malformed blood vessels being treated.

A staged microsurgical treatment method was adopted after a comprehensive evaluation based on a series of examinations and the surgeon’s experience. In the first stage of surgery, the right occipital artery, posterior auricular artery/vein, arterialized fistula behind the superficial temporal artery, and fistula of the superficial temporal artery were blocked and ligated first. The right superficial temporal artery and vein were left as the main supply arteries and drainage veins to ensure the local blood supply of the scalp. The volume of the vascular malformations was significantly reduced subcutaneously, and the plexiform dilated blood vessels around the vascular malformations in the right parietal partially returned to normal a month later, which indicated that the first stage of the surgery was effective. In the second stage of the surgery, an “S”-shaped incision was adopted to remove the subcutaneous vascular malformations thoroughly. The top side of the “S”-shaped incision can further block the plexiform dilated blood vessels in the subcutaneous area around the vascular malformations in the right parietal. The middle part of the “S”-shaped incision can block the fistula of the right occipital artery, and the occipital side of the “S”-shaped incision can further block the blood supply to the vascular malformation in the subcutaneous region of the right occipital artery. Intraoperative bleeding could not only be reduced but also be used to ensure blood supply from the superficial temporal artery to the scalp through this surgical incision. It was found that the scalp incision healed well a month later, and there was no recurrence of the sAVF after 2 years of follow-up, which was considered cured. This article provides a feasible solution for the treatment of a complex sAVF that contained C-type sAVF and CA through staged microsurgery. If there are some shortcomings, may we ask our peer experts to give corrections.

## 5 Conclusion

For complex sAVFs, especially C-type sAVFs combined with CA, the first treatment can include interventional endovascular embolization or microsurgical ligation of blood supply vessels to reduce the volume of the lesion. Then, vascular malformations can be completely resected through personalized surgical incisions designed based on the size, range, number, and location of blood supply arteries and drainage veins of the patient’s arteriovenous fistula.

## Data Availability

The raw data supporting the conclusions of this article will be made available by the authors, without undue reservation.

## References

[1] Zheng J Guo Z Zhang X Sun X. Intravascular embolization versus surgical resection for patients with scalp arteriovenous fistula. Chin Neurosurg J. (2019) 5:3. doi: 10.1186/s41016-018-0148-1 32922903 PMC7398378

[2] Alfaro AJQ Ortíz AFH Mejia JA Ortegon JDC Gutierrez LC Tovar CAD. Traumatic scalp arteriovenous fistula post capillary implantation successfully treated using PHIL embolic agent. Surg Neurol Int. (2023) 14:12. doi: 10.25259/SNI_1002_2022 36751445 PMC9899462

[3] Walker GB Wang AP Hadwen J Erdenebold UE Bebedjian R Sullivan P. Direct puncture of the superficial temporal artery in embolization of a scalp Arteriovenous fistula: A case report. Neurointervention. (2023) 18:67–71. doi: 10.5469/neuroint.2022.00465 36717084 PMC9986351

[4] Biegaj E Rutkowska-Zimirska J Radzymińska-Maliszewska M Zaremba A Pniewski J. Arteriovenous fistula of superficial temporal vessels. Folia Morphol (Warsz). (2019) 78:879–82. doi: 10.5603/FM.a2019.0016 30816554

[5] Kojima D Akamatsu Y Fujimoto K Oikawa K Kashimura H Kubo Y. Utility of manual venous compression during transvenous Onyx injection for a scalp arteriovenous fistula: illustrative case. J Neurosurg Case Lessons. (2022) 4:1–5. doi: 10.3171/CASE22317 PMC9624155 36317235

[6] Bekele K Gezahegn H. Traumatic Arteriovenous fistula of superficial temporal vessel: A rare case report. Int Med Case Rep J. (2021) 14:483–5. doi: 10.2147/IMCRJ.S313259 PMC8289310 34290533

[7] Yokouchi T Iwabuchi S Tomiyama A Samejima H Ogata N Goto K. Embolization of scalp AVF. Interv Neuroradiol. (1999) 5 Suppl 1:121–6. doi: 10.1177/15910199990050S122 20670552

[8] Janapareddy KK Peters NJ Samujh R Kumar A Malik MA Chabbra A. A fix for a scalp varix! A rare case of cirsoid aneurysm in a child. J Indian Assoc Pediatr Surg. (2022) 27:100–2. doi: 10.4103/jiaps.JIAPS_273_20 PMC8853608 35261523

[9] Ni W Tian Y Gu Y Mao Y. Transvenous endovascular treatment for scalp arteriovenous fistulas: results with combined use of onyx and coils. World Neurosurg. (2017) 107:692–7. doi: 10.1016/j.wneu.2017.08.056 28838879

[10] Hemperly S McClain R Lountzis N Purcell SM. Scalp arteriovenous fistula with intracranial communication. Cutis. (2020) 106:E9–E10. doi: 10.12788/cutis 33465200

[11] Senoglu M Yasim A Gokce M Senoglu N. Nontraumatic scalp arteriovenous fistula in an adult: technical report on an illustrative case. Surg Neurol. (2008) 70:194–7. doi: 10.1016/j.surneu.2007.04.018 18291475

[12] Sofela A Osunronbi T Hettige S. Scalp cirsoid aneurysms: case illustration and systematic review of literature. Neurosurgery. (2020) 86:E98–E107. doi: 10.1093/neuros/nyz303 31384940

[13] Kumar A Ahuja CK Khandelwal N Bakshi JB. Cirsoid aneurysm of the right pre-auricular region: an unusual cause of tinnitus managed by endovascular glue embolisation. J Laryngol Otol. (2012) 126:923–7. doi: 10.1017/S0022215112001466 22874530

[14] Albuquerque Sousa LH Maranha Gatto LA Demartini Junior Z Koppe GL. Scalp Cirsoid aneurysm: an updated systematic literature review and an illustrative case report. World Neurosurg. (2018) 119:416–27. doi: 10.1016/j.wneu.2018.08.098 30149169

